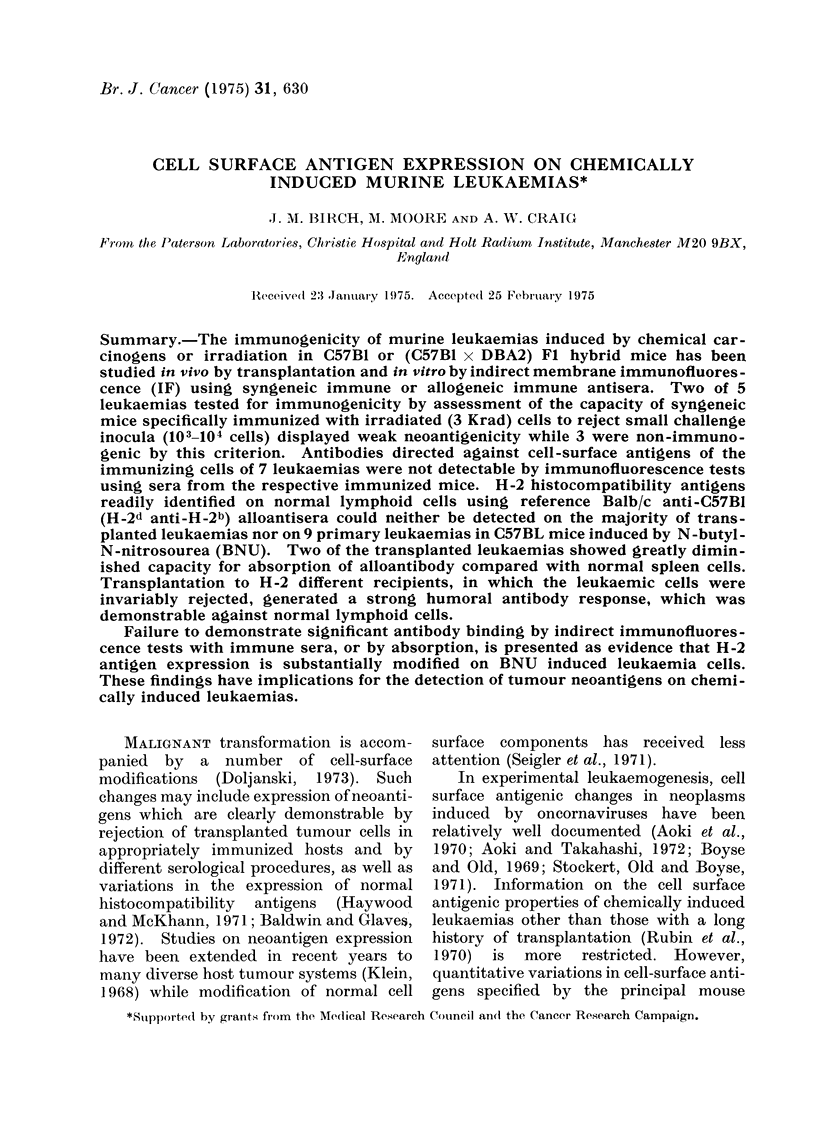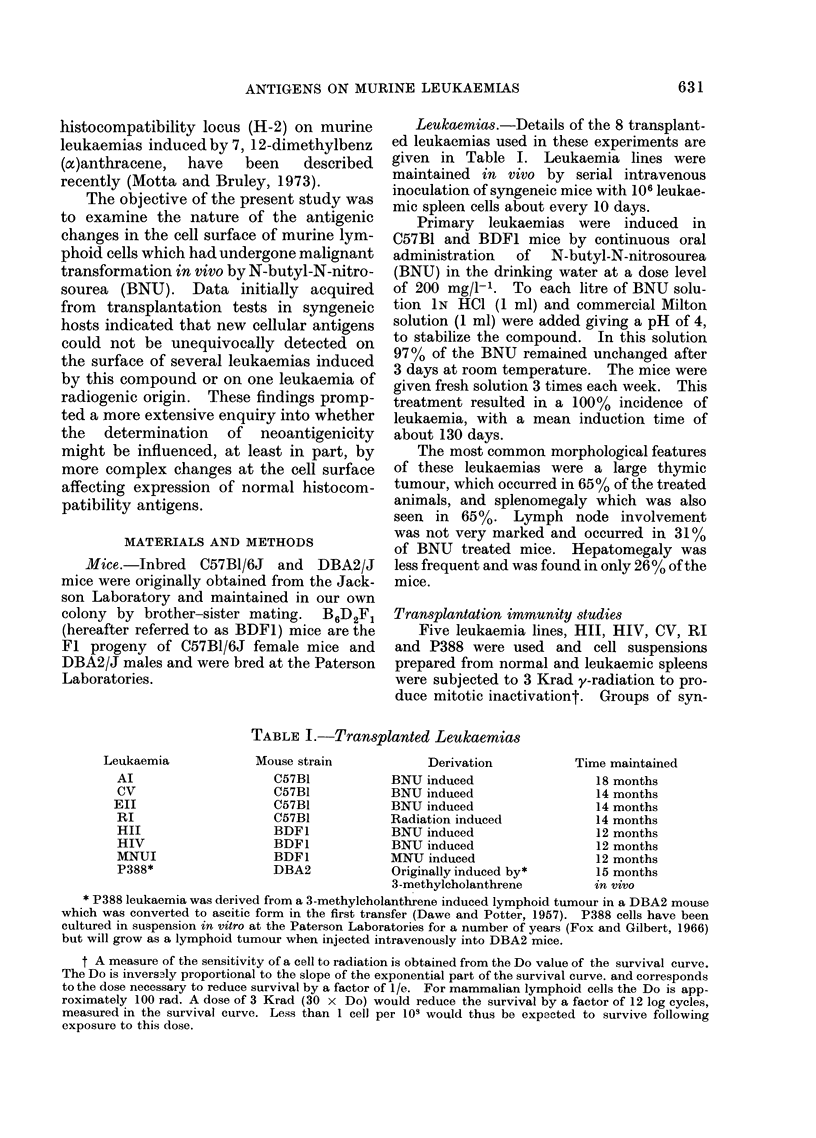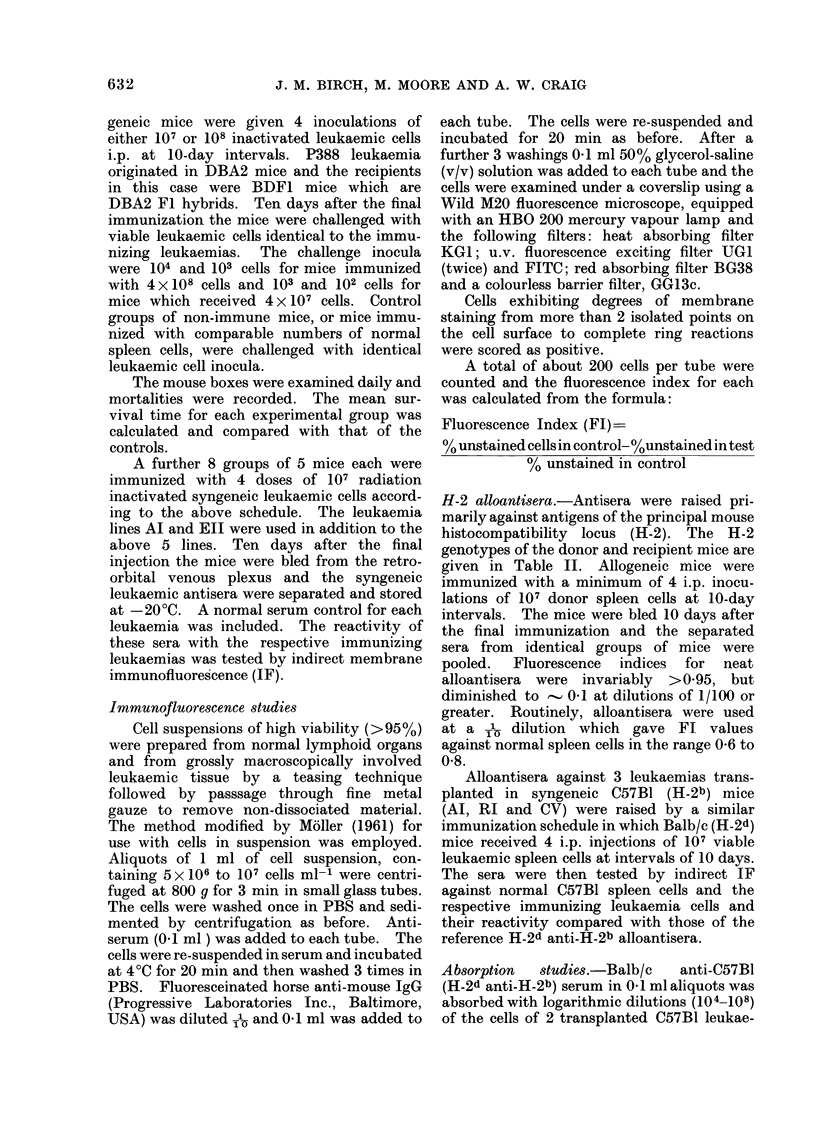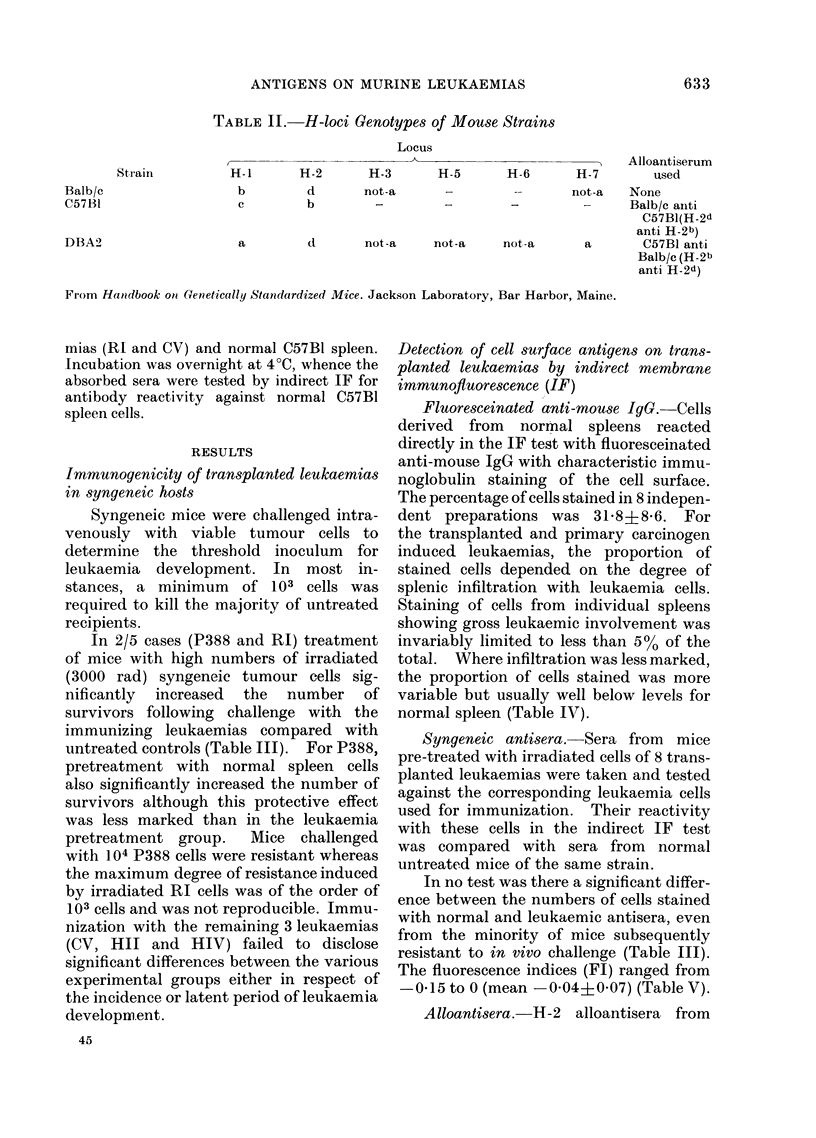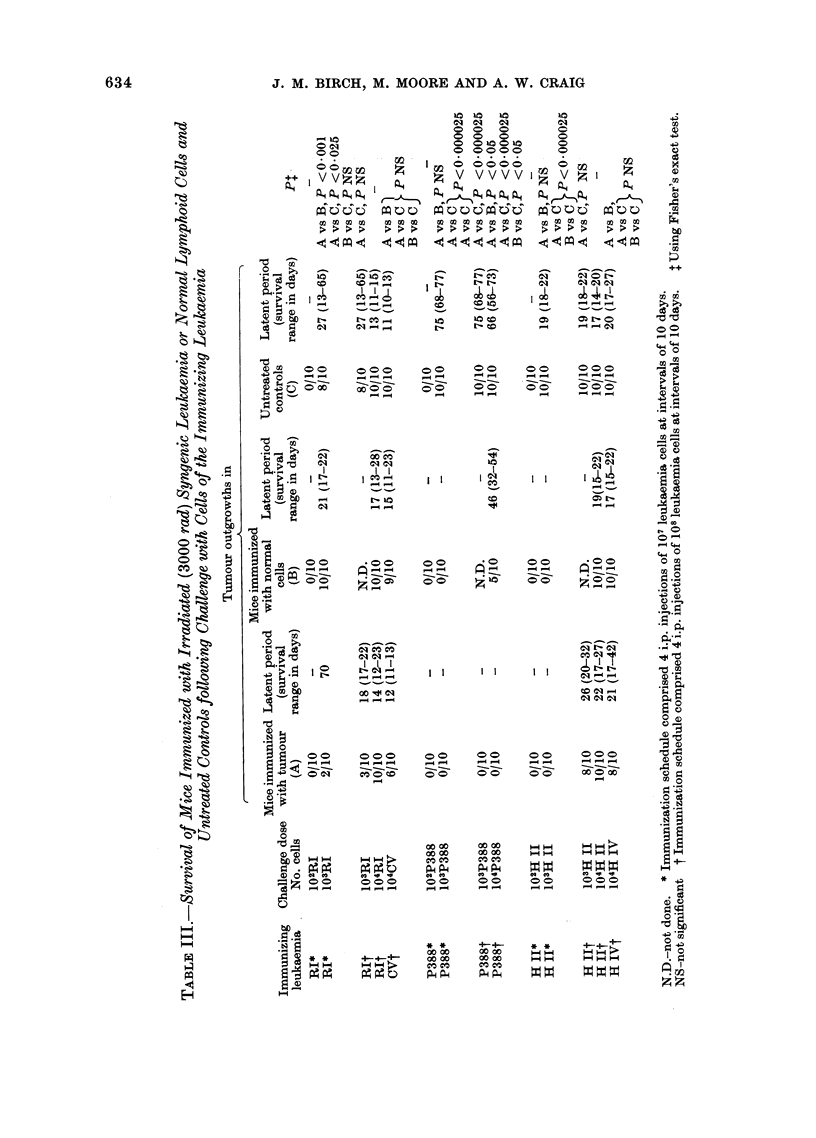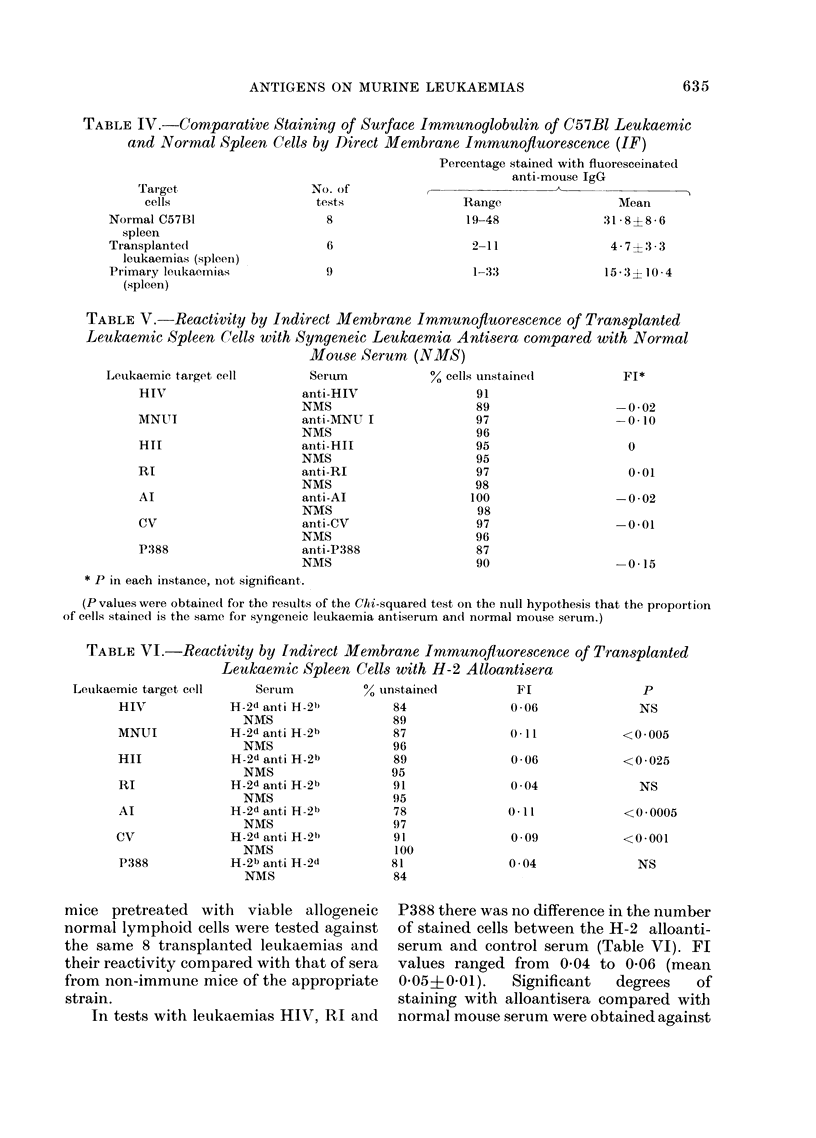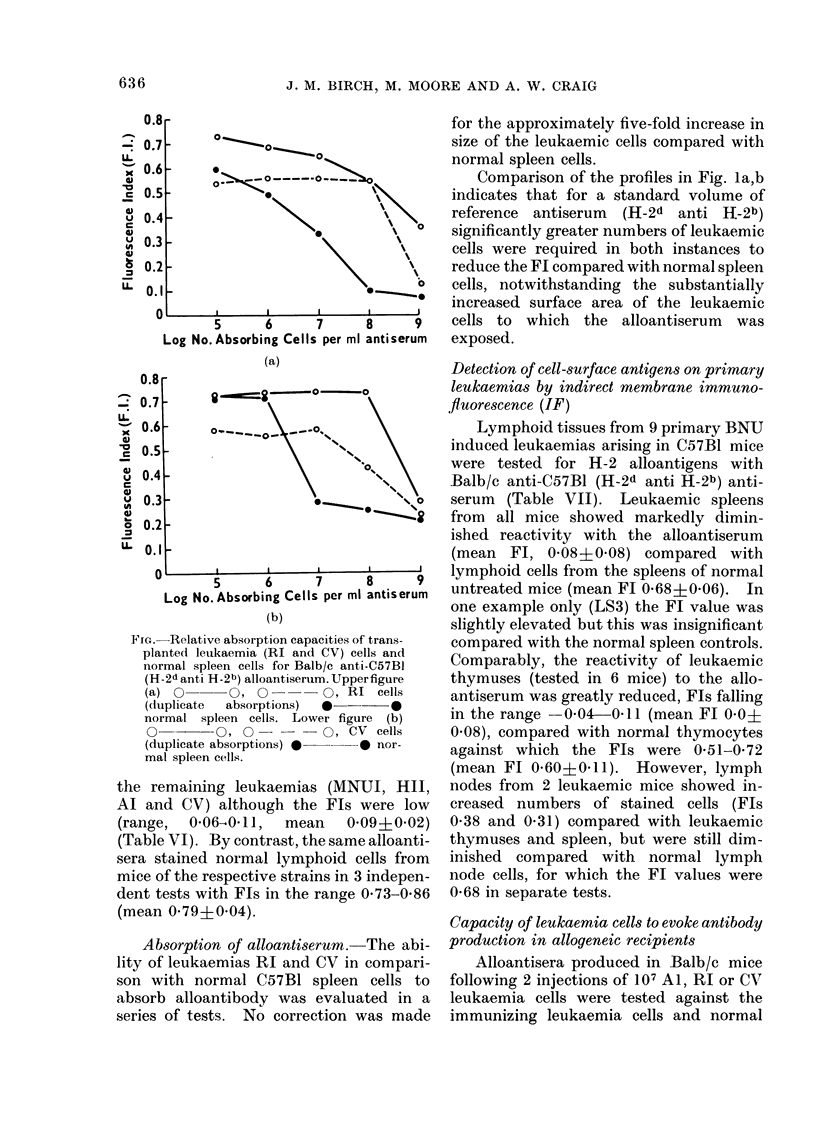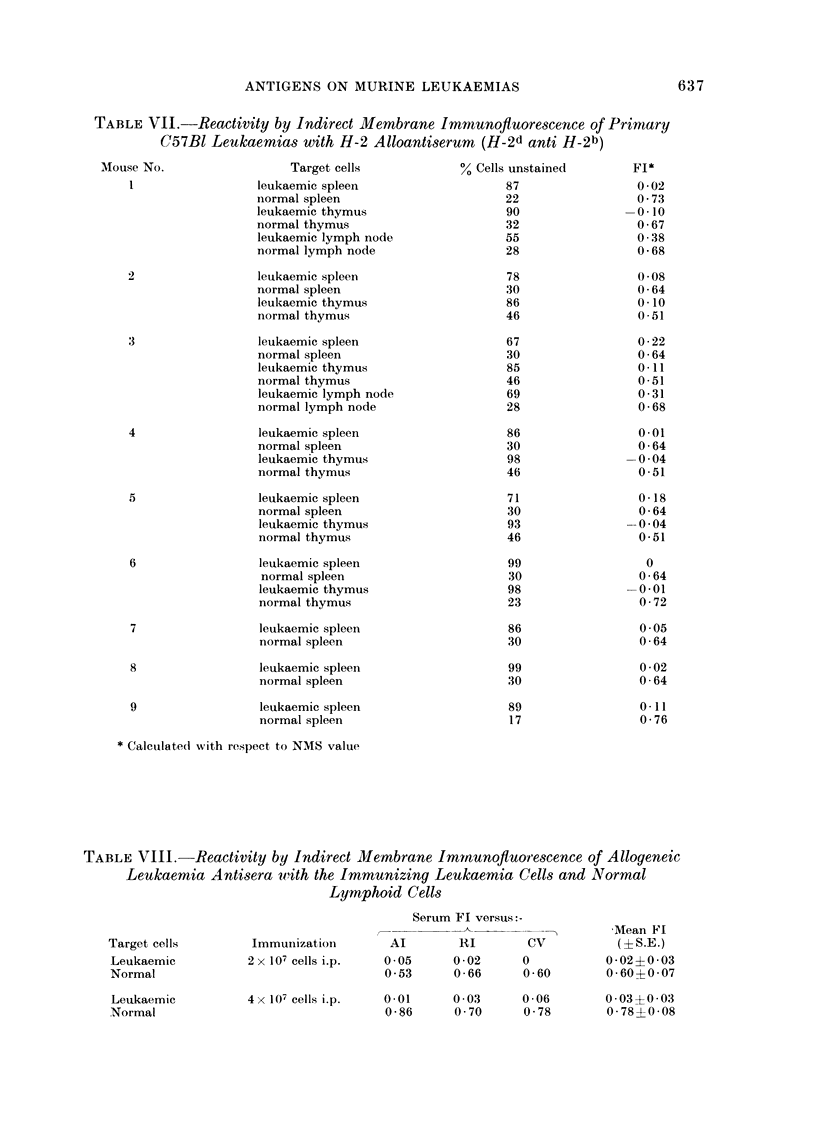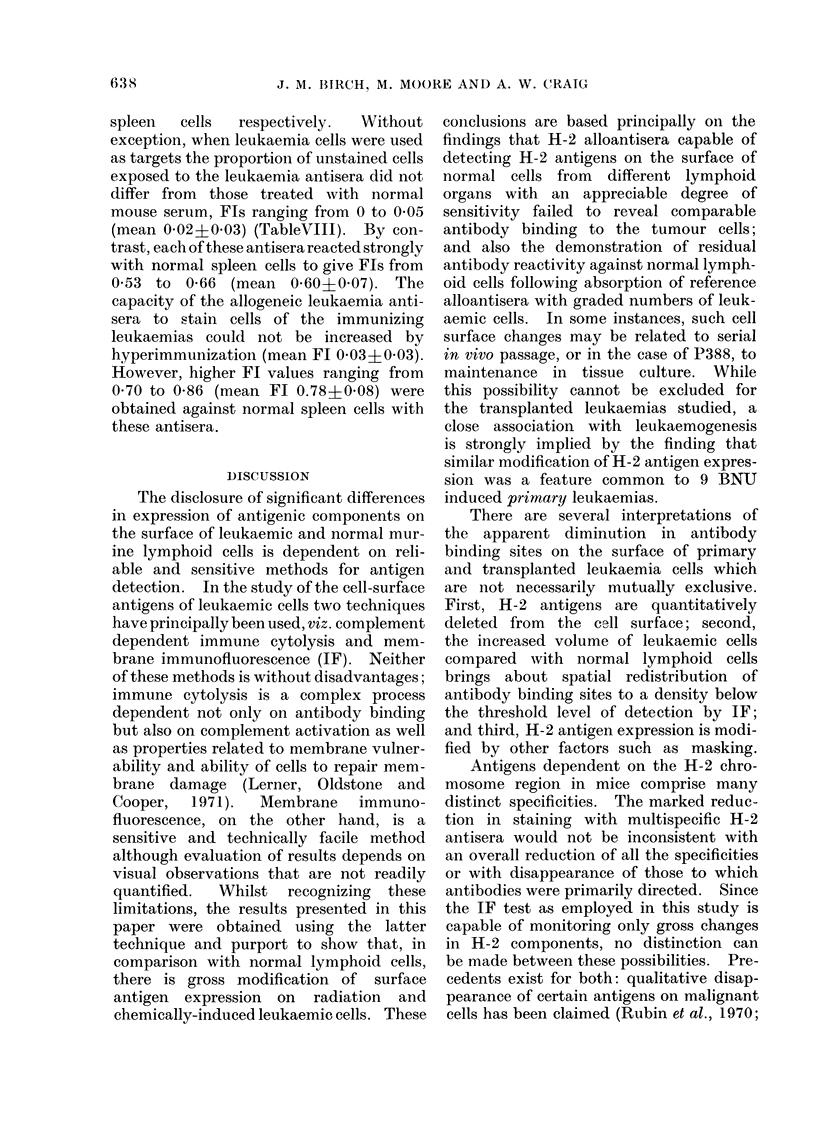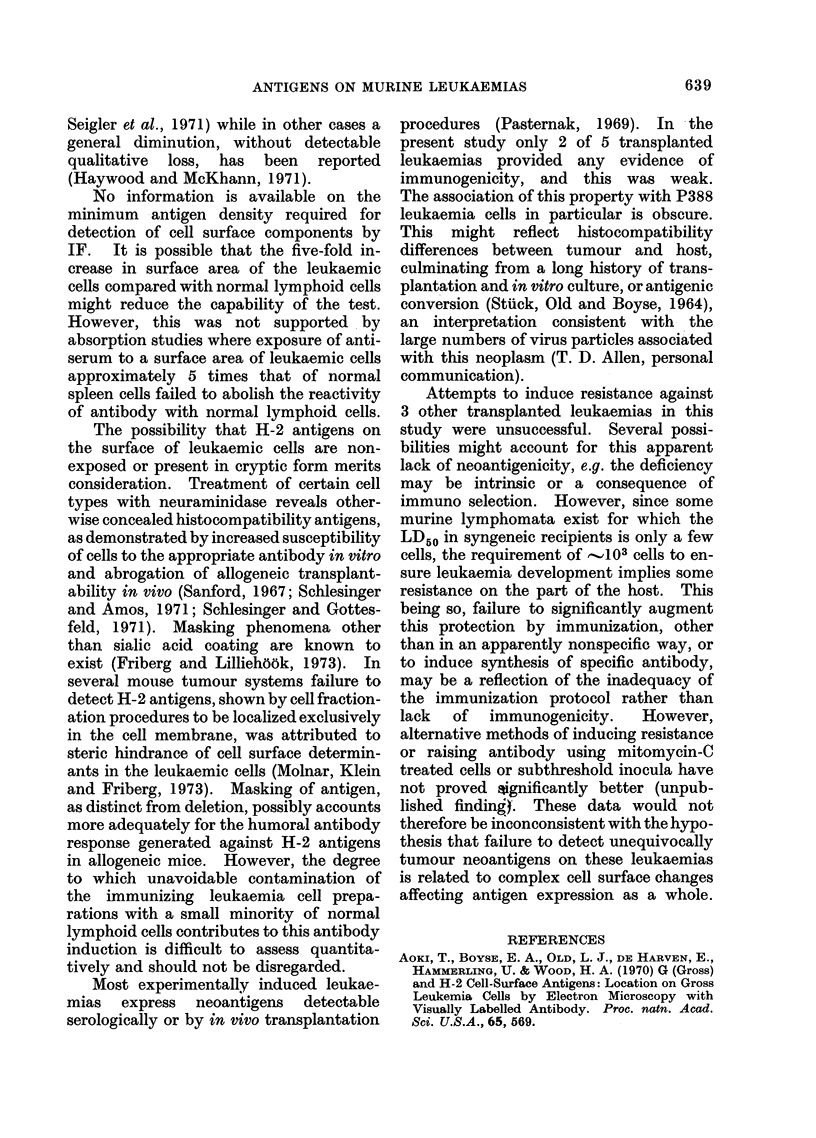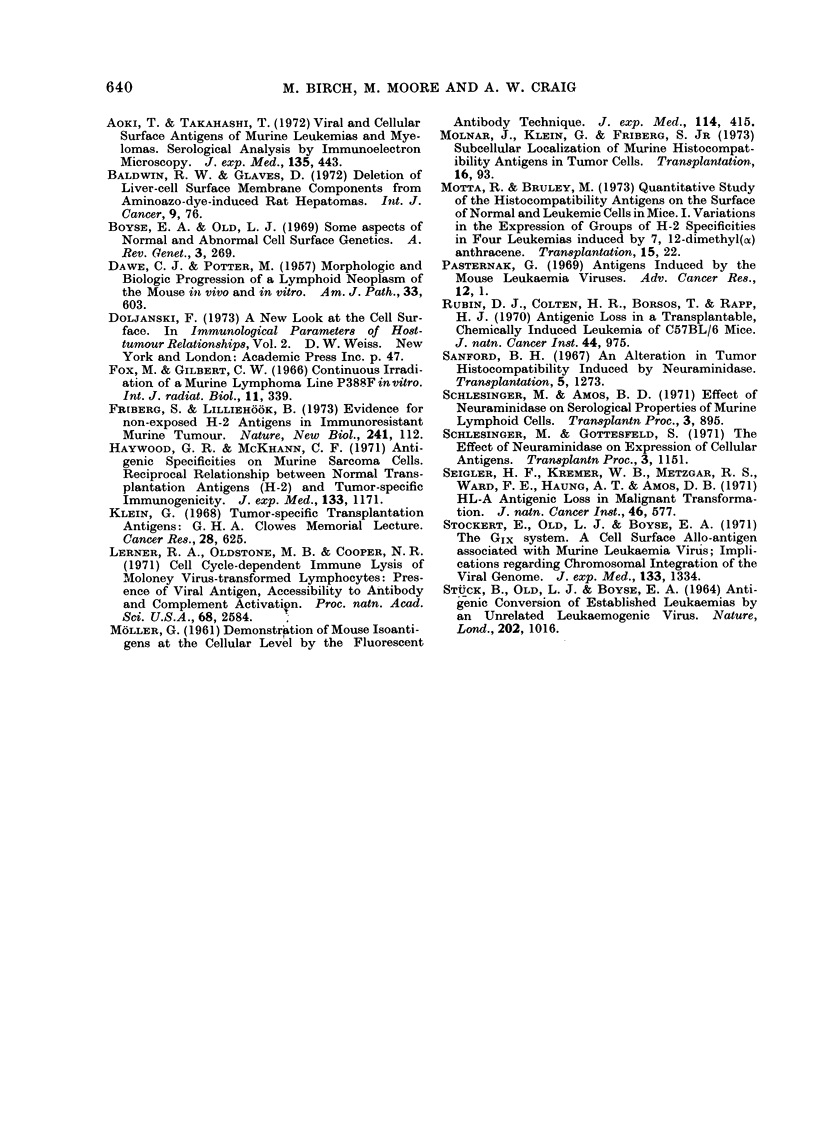# Cell surface antigen expression on chemically induced murine leukaemias.

**DOI:** 10.1038/bjc.1975.109

**Published:** 1975-06

**Authors:** J. M. Birch, M. Moore, A. W. Craig

## Abstract

The immunogenicity of murine leukaemias induced by chemical carcinogens or irradiation in C57Bl or (C57Bl times DBA2) F1 hybrid mice has been studied in vivo by transplantation and in vitro by indirect membrane immunofluorescence (IF) using syngeneic immune or allogeneic immune antisera. Two of 5 leukaemias tested for immunogenicity by assessment of the capacity of syngeneic mice specifically immunized with irradiated (3 Krad) cells to reject small challenge inocula (10(3)-10(4) cells) displayed weak neoantigenicity while 3 were non-immunogenic by this criterion. Antibodies directed against cell-surface antigens of the immunizing cells of 7 leukaemias were not detectable by immunofluorescence tests using sera from the respective immunized mice. H-2 histocompatibility antigens readily identified on normal lymphoid cells using reference Balb/c anti-C57Bl (H-2d anti-H-2b) alloantisera could neither be detected on the majority of transplanted leukaemias nor on 9 primary leukaemias in C57Bl mice induced by N-butyl-N-nitrosourea (BNU). Two of the transplanted leukaemias showed greatly diminished capacity for absorption of alloantibody compared with normal spleen cells. Transplantation to H-2 different recipients, in which the leukaemic cells were invariably rejected, generated a strong humoral antibody response, which was demonstrable against normal lymphoid cells. Failure to demonstrate significant antibody binding by indirect immunofluroescence tests with immune sera, or by absorption, is presented as evidency that H-2 antigen expression is substantially modified on BNU induced leukaemia cells. These findings have implications for the detection of tumour neoantigens on chemically induced leukaemias.


*Br. J. Cancer* (1975) **31**, 630

### CELL SURFACE ANTIGEN EXPRESSION ON CHEMICALLY INDUCED MURINE LEUKAEMIAS*

J. M. BIRCH, M. MOORE AND A. W. CRAIG

*From the Paterson Laboratories, Christie Hospital and Holt Radium Institute, Manchester M20 9BX, England*

Received 23 January 1975. Accepted 25 February 1975

**Summary.—The immunogenicity of murine leukaemias induced by chemical carcinogens or irradiation in C57Bl or (C57Bl × DBA2) F1 hybrid mice has been studied *in vivo* by transplantation and *in vitro* by indirect membrane immunofluorescence (IF) using syngeneic immune or allogeneic immune antisera. Two of 5 leukaemias tested for immunogenicity by assessment of the capacity of syngeneic mice specifically immunized with irradiated (3 Krad) cells to reject small challenge inocula ($10^3$–$10^4$ cells) displayed weak neoantigenicity while 3 were non-immunogenic by this criterion. Antibodies directed against cell-surface antigens of the immunizing cells of 7 leukaemias were not detectable by immunofluorescence tests using sera from the respective immunized mice. H-2 histocompatibility antigens readily identified on normal lymphoid cells using reference Balb/c anti-C57Bl ($H\text{-}2^d$ anti-$H\text{-}2^b$) alloantisera could neither be detected on the majority of transplanted leukaemias nor on 9 primary leukaemias in C57BL mice induced by N-butyl-N-nitrosourea (BNU). Two of the transplanted leukaemias showed greatly diminished capacity for absorption of alloantibody compared with normal spleen cells. Transplantation to H-2 different recipients, in which the leukaemic cells were invariably rejected, generated a strong humoral antibody response, which was demonstrable against normal lymphoid cells.**

**Failure to demonstrate significant antibody binding by indirect immunofluorescence tests with immune sera, or by absorption, is presented as evidence that H-2 antigen expression is substantially modified on BNU induced leukaemia cells. These findings have implications for the detection of tumour neoantigens on chemically induced leukaemias.**

MALIGNANT transformation is accompanied by a number of cell-surface modifications (Doljanski, 1973). Such changes may include expression of neoantigens which are clearly demonstrable by rejection of transplanted tumour cells in appropriately immunized hosts and by different serological procedures, as well as variations in the expression of normal histocompatibility antigens (Haywood and McKhann, 1971; Baldwin and Glaves, 1972). Studies on neoantigen expression have been extended in recent years to many diverse host tumour systems (Klein, 1968) while modification of normal cell surface components has received less attention (Seigler *et al.*, 1971).

In experimental leukaemogenesis, cell surface antigenic changes in neoplasms induced by oncornaviruses have been relatively well documented (Aoki *et al.*, 1970; Aoki and Takahashi, 1972; Boyse and Old, 1969; Stockert, Old and Boyse, 1971). Information on the cell surface antigenic properties of chemically induced leukaemias other than those with a long history of transplantation (Rubin *et al.*, 1970) is more restricted. However, quantitative variations in cell-surface antigens specified by the principal mouse

*Supported by grants from the Medical Research Council and the Cancer Research Campaign.

histocompatibility locus (H-2) on murine leukaemias induced by 7, 12-dimethylbenz ($\alpha$)anthracene, have been described recently (Motta and Bruley, 1973).

The objective of the present study was to examine the nature of the antigenic changes in the cell surface of murine lymphoid cells which had undergone malignant transformation *in vivo* by N-butyl-N-nitrosourea (BNU). Data initially acquired from transplantation tests in syngeneic hosts indicated that new cellular antigens could not be unequivocally detected on the surface of several leukaemias induced by this compound or on one leukaemia of radiogenic origin. These findings prompted a more extensive enquiry into whether the determination of neoantigenicity might be influenced, at least in part, by more complex changes at the cell surface affecting expression of normal histocompatibility antigens.

### MATERIALS AND METHODS

*Mice.*—Inbred C57Bl/6J and DBA2/J mice were originally obtained from the Jackson Laboratory and maintained in our own colony by brother–sister mating. $B_6D_2F_1$ (hereafter referred to as BDF1) mice are the F1 progeny of C57Bl/6J female mice and DBA2/J males and were bred at the Paterson Laboratories.

*Leukaemias.*—Details of the 8 transplanted leukaemias used in these experiments are given in Table I. Leukaemia lines were maintained *in vivo* by serial intravenous inoculation of syngeneic mice with $10^6$ leukaemic spleen cells about every 10 days.

Primary leukaemias were induced in C57Bl and BDF1 mice by continuous oral administration of N-butyl-N-nitrosourea (BNU) in the drinking water at a dose level of 200 mg/l$^{-1}$. To each litre of BNU solution 1N HCl (1 ml) and commercial Milton solution (1 ml) were added giving a pH of 4, to stabilize the compound. In this solution 97% of the BNU remained unchanged after 3 days at room temperature. The mice were given fresh solution 3 times each week. This treatment resulted in a 100% incidence of leukaemia, with a mean induction time of about 130 days.

The most common morphological features of these leukaemias were a large thymic tumour, which occurred in 65% of the treated animals, and splenomegaly which was also seen in 65%. Lymph node involvement was not very marked and occurred in 31% of BNU treated mice. Hepatomegaly was less frequent and was found in only 26% of the mice.

### *Transplantation immunity studies*

Five leukaemia lines, HII, HIV, CV, RI and P388 were used and cell suspensions prepared from normal and leukaemic spleens were subjected to 3 Krad $\gamma$-radiation to produce mitotic inactivation†. Groups of syn-

TABLE I.—*Transplanted Leukaemias*

| Leukaemia | Mouse strain | Derivation | Time maintained |
|---|---|---|---|
| AI | C57Bl | BNU induced | 18 months |
| CV | C57Bl | BNU induced | 14 months |
| EII | C57Bl | BNU induced | 14 months |
| RI | C57Bl | Radiation induced | 14 months |
| HII | BDF1 | BNU induced | 12 months |
| HIV | BDF1 | BNU induced | 12 months |
| MNUI | BDF1 | MNU induced | 12 months |
| P388* | DBA2 | Originally induced by* 3-methylcholanthrene | 15 months *in vivo* |

* P388 leukaemia was derived from a 3-methylcholanthrene induced lymphoid tumour in a DBA2 mouse which was converted to ascitic form in the first transfer (Dawe and Potter, 1957). P388 cells have been cultured in suspension *in vitro* at the Paterson Laboratories for a number of years (Fox and Gilbert, 1966) but will grow as a lymphoid tumour when injected intravenously into DBA2 mice.

† A measure of the sensitivity of a cell to radiation is obtained from the Do value of the survival curve. The Do is inversely proportional to the slope of the exponential part of the survival curve. and corresponds to the dose necessary to reduce survival by a factor of 1/e. For mammalian lymphoid cells the Do is approximately 100 rad. A dose of 3 Krad (30 × Do) would reduce the survival by a factor of 12 log cycles, measured in the survival curve. Less than 1 cell per $10^8$ would thus be expected to survive following exposure to this dose.

geneic mice were given 4 inoculations of either $10^7$ or $10^8$ inactivated leukaemic cells i.p. at 10-day intervals. P388 leukaemia originated in DBA2 mice and the recipients in this case were BDF1 mice which are DBA2 F1 hybrids. Ten days after the final immunization the mice were challenged with viable leukaemic cells identical to the immunizing leukaemias. The challenge inocula were $10^4$ and $10^3$ cells for mice immunized with $4 \times 10^8$ cells and $10^3$ and $10^2$ cells for mice which received $4 \times 10^7$ cells. Control groups of non-immune mice, or mice immunized with comparable numbers of normal spleen cells, were challenged with identical leukaemic cell inocula.

The mouse boxes were examined daily and mortalities were recorded. The mean survival time for each experimental group was calculated and compared with that of the controls.

A further 8 groups of 5 mice each were immunized with 4 doses of $10^7$ radiation inactivated syngeneic leukaemic cells according to the above schedule. The leukaemia lines AI and EII were used in addition to the above 5 lines. Ten days after the final injection the mice were bled from the retro-orbital venous plexus and the syngeneic leukaemic antisera were separated and stored at $-20$°C. A normal serum control for each leukaemia was included. The reactivity of these sera with the respective immunizing leukaemias was tested by indirect membrane immunofluorescence (IF).

*Immunofluorescence studies*

Cell suspensions of high viability (>95%) were prepared from normal lymphoid organs and from grossly macroscopically involved leukaemic tissue by a teasing technique followed by passsage through fine metal gauze to remove non-dissociated material. The method modified by Möller (1961) for use with cells in suspension was employed. Aliquots of 1 ml of cell suspension, containing $5 \times 10^6$ to $10^7$ cells $ml^{-1}$ were centrifuged at 800 *g* for 3 min in small glass tubes. The cells were washed once in PBS and sedimented by centrifugation as before. Antiserum (0·1 ml ) was added to each tube. The cells were re-suspended in serum and incubated at 4°C for 20 min and then washed 3 times in PBS. Fluoresceinated horse anti-mouse IgG (Progressive Laboratories Inc., Baltimore, USA) was diluted $\frac{1}{10}$ and 0·1 ml was added to each tube. The cells were re-suspended and incubated for 20 min as before. After a further 3 washings 0·1 ml 50% glycerol-saline (v/v) solution was added to each tube and the cells were examined under a coverslip using a Wild M20 fluorescence microscope, equipped with an HBO 200 mercury vapour lamp and the following filters: heat absorbing filter KG1; u.v. fluorescence exciting filter UG1 (twice) and FITC; red absorbing filter BG38 and a colourless barrier filter, GG13c.

Cells exhibiting degrees of membrane staining from more than 2 isolated points on the cell surface to complete ring reactions were scored as positive.

A total of about 200 cells per tube were counted and the fluorescence index for each was calculated from the formula:

$$\text{Fluorescence Index (FI)} = \frac{\%\,\text{unstained cells in control} - \%\,\text{unstained in test}}{\%\,\text{unstained in control}}$$

*H-2 alloantisera.*—Antisera were raised primarily against antigens of the principal mouse histocompatibility locus (H-2). The H-2 genotypes of the donor and recipient mice are given in Table II. Allogeneic mice were immunized with a minimum of 4 i.p. inoculations of $10^7$ donor spleen cells at 10-day intervals. The mice were bled 10 days after the final immunization and the separated sera from identical groups of mice were pooled. Fluorescence indices for neat alloantisera were invariably $>0·95$, but diminished to $\sim 0·1$ at dilutions of 1/100 or greater. Routinely, alloantisera were used at a $\frac{1}{10}$ dilution which gave FI values against normal spleen cells in the range 0·6 to 0·8.

Alloantisera against 3 leukaemias transplanted in syngeneic C57Bl (H-$2^b$) mice (AI, RI and CV) were raised by a similar immunization schedule in which Balb/c (H-$2^d$) mice received 4 i.p. injections of $10^7$ viable leukaemic spleen cells at intervals of 10 days. The sera were then tested by indirect IF against normal C57Bl spleen cells and the respective immunizing leukaemia cells and their reactivity compared with those of the reference H-$2^d$ anti-H-$2^b$ alloantisera.

*Absorption studies.*—Balb/c anti-C57Bl (H-$2^d$ anti-H-$2^b$) serum in 0·1 ml aliquots was absorbed with logarithmic dilutions ($10^4$–$10^8$) of the cells of 2 transplanted C57Bl leukae-

TABLE II.—*H-loci Genotypes of Mouse Strains*

| Strain | Locus H-1 | H-2 | H-3 | H-5 | H-6 | H-7 | Alloantiserum used |
|---|---|---|---|---|---|---|---|
| Balb/c | b | d | not-a | – | – | not-a | None |
| C57Bl | c | b | – | – | – | – | Balb/c anti C57Bl(H-$2^d$ anti H-$2^b$) |
| DBA2 | a | d | not-a | not-a | not-a | a | C57Bl anti Balb/c (H-$2^b$ anti H-$2^d$) |

From *Handbook on Genetically Standardized Mice*. Jackson Laboratory, Bar Harbor, Maine.

mias (RI and CV) and normal C57Bl spleen. Incubation was overnight at 4°C, whence the absorbed sera were tested by indirect IF for antibody reactivity against normal C57Bl spleen cells.

### RESULTS

### *Immunogenicity of transplanted leukaemias in syngeneic hosts*

Syngeneic mice were challenged intravenously with viable tumour cells to determine the threshold inoculum for leukaemia development. In most instances, a minimum of $10^3$ cells was required to kill the majority of untreated recipients.

In 2/5 cases (P388 and RI) treatment of mice with high numbers of irradiated (3000 rad) syngeneic tumour cells significantly increased the number of survivors following challenge with the immunizing leukaemias compared with untreated controls (Table III). For P388, pretreatment with normal spleen cells also significantly increased the number of survivors although this protective effect was less marked than in the leukaemia pretreatment group. Mice challenged with $10^4$ P388 cells were resistant whereas the maximum degree of resistance induced by irradiated RI cells was of the order of $10^3$ cells and was not reproducible. Immunization with the remaining 3 leukaemias (CV, HII and HIV) failed to disclose significant differences between the various experimental groups either in respect of the incidence or latent period of leukaemia development.

### *Detection of cell surface antigens on transplanted leukaemias by indirect membrane immunofluorescence (IF)*

*Fluoresceinated anti-mouse IgG*.—Cells derived from normal spleens reacted directly in the IF test with fluoresceinated anti-mouse IgG with characteristic immunoglobulin staining of the cell surface. The percentage of cells stained in 8 independent preparations was $31{\cdot}8 \pm 8{\cdot}6$. For the transplanted and primary carcinogen induced leukaemias, the proportion of stained cells depended on the degree of splenic infiltration with leukaemia cells. Staining of cells from individual spleens showing gross leukaemic involvement was invariably limited to less than 5% of the total. Where infiltration was less marked, the proportion of cells stained was more variable but usually well below levels for normal spleen (Table IV).

*Syngeneic antisera*.—Sera from mice pre-treated with irradiated cells of 8 transplanted leukaemias were taken and tested against the corresponding leukaemia cells used for immunization. Their reactivity with these cells in the indirect IF test was compared with sera from normal untreated mice of the same strain.

In no test was there a significant difference between the numbers of cells stained with normal and leukaemic antisera, even from the minority of mice subsequently resistant to *in vivo* challenge (Table III). The fluorescence indices (FI) ranged from $-0{\cdot}15$ to 0 (mean $-0{\cdot}04 \pm 0{\cdot}07$) (Table V).

*Alloantisera*.—H-2 alloantisera from

TABLE III.—*Survival of Mice Immunized with Irradiated* (3000 *rad*) *Syngenic Leukaemia or Normal Lymphoid Cells and Untreated Controls following Challenge with Cells of the Immunizing Leukaemia*

| | | Tumour outgrowths in | | | | | | |
|---|---|---|---|---|---|---|---|---|
| Immunizing leukaemia | Challenge dose No. cells | Mice immunized with tumour (A) | Latent period (survival range in days) | Mice immunized with normal cells (B) | Latent period (survival range in days) | Untreated controls (C) | Latent period (survival range in days) | P‡ |
| RI* | $10^2$RI | 0/10 | – | 0/10 | – | 0/10 | – | – |
| RI* | $10^3$RI | 2/10 | 70 | 10/10 | 21 (17–22) | 8/10 | 27 (13–65) | A vs B, $P <0\cdot001$; A vs C, $P <0\cdot025$; B vs C, $P$ NS |
| RI† | $10^3$RI | 3/10 | 18 (17–22) | N.D. | – | 8/10 | 27 (13–65) | A vs C, $P$ NS |
| RI† | $10^4$RI | 10/10 | 14 (12–23) | 10/10 | 17 (13–28) | 10/10 | 13 (11–15) | – |
| CV† | $10^4$CV | 6/10 | 12 (11–13) | 9/10 | 15 (11–23) | 10/10 | 11 (10–13) | A vs B, A vs C, B vs C } $P$ NS |
| P388* | $10^2$P388 | 0/10 | – | 0/10 | – | 0/10 | – | – |
| P388* | $10^3$P388 | 0/10 | – | 0/10 | – | 10/10 | 75 (68–77) | A vs B, $P$ NS; A vs C, A vs C } $P<0\cdot000025$ |
| P388† | $10^3$P388 | 0/10 | – | N.D. | – | 10/10 | 75 (68–77) | A vs C, $P <0\cdot000025$ |
| P388† | $10^4$P388 | 0/10 | – | 5/10 | 46 (32–54) | 10/10 | 66 (56–73) | A vs B, $P <0\cdot05$; A vs C, $P <0\cdot000025$; B vs C, $P <0\cdot05$ |
| H II* | $10^2$H II | 0/10 | – | 0/10 | – | 0/10 | – | – |
| H II* | $10^3$H II | 0/10 | – | 0/10 | – | 10/10 | 19 (18–22) | A vs B, $P$ NS; A vs C, B vs C } $P<0\cdot000025$ |
| H II† | $10^3$H II | 8/10 | 26 (20–32) | N.D. | – | 10/10 | 19 (18–22) | A vs C, $P$ NS |
| H II† | $10^4$H II | 10/10 | 22 (17–27) | 10/10 | 19(15–22) | 10/10 | 17 (14–20) | – |
| H IV† | $10^4$H IV | 8/10 | 21 (17–42) | 10/10 | 17 (15–22) | 10/10 | 20 (17–27) | A vs B, A vs C, B vs C } $P$ NS |

N.D.-not done. * Immunization schedule comprised 4 i.p. injections of $10^7$ leukaemia cells at intervals of 10 days.

NS-not significant † Immunization schedule comprised 4 i.p. injections of $10^8$ leukaemia cells at intervals of 10 days. ‡ Using Fisher's exact test.

TABLE IV.—*Comparative Staining of Surface Immunoglobulin of C57Bl Leukaemic and Normal Spleen Cells by Direct Membrane Immunofluorescence (IF)*

| Target cells | No. of tests | Percentage stained with fluoresceinated anti-mouse IgG | |
|---|---|---|---|
| | | Range | Mean |
| Normal C57Bl spleen | 8 | 19–48 | $31 \cdot 8 \pm 8 \cdot 6$ |
| Transplanted leukaemias (spleen) | 6 | 2–11 | $4 \cdot 7 \pm 3 \cdot 3$ |
| Primary leukaemias (spleen) | 9 | 1–33 | $15 \cdot 3 \pm 10 \cdot 4$ |

TABLE V.—*Reactivity by Indirect Membrane Immunofluorescence of Transplanted Leukaemic Spleen Cells with Syngeneic Leukaemia Antisera compared with Normal Mouse Serum (NMS)*

| Leukaemic target cell | Serum | % cells unstained | FI* |
|---|---|---|---|
| HIV | anti-HIV | 91 | |
| | NMS | 89 | −0·02 |
| MNUI | anti-MNU I | 97 | −0·10 |
| | NMS | 96 | |
| HII | anti-HII | 95 | 0 |
| | NMS | 95 | |
| RI | anti-RI | 97 | 0·01 |
| | NMS | 98 | |
| AI | anti-AI | 100 | −0·02 |
| | NMS | 98 | |
| CV | anti-CV | 97 | −0·01 |
| | NMS | 96 | |
| P388 | anti-P388 | 87 | |
| | NMS | 90 | −0·15 |

* *P* in each instance, not significant.

(*P* values were obtained for the results of the *Chi*-squared test on the null hypothesis that the proportion of cells stained is the same for syngeneic leukaemia antiserum and normal mouse serum.)

TABLE VI.—*Reactivity by Indirect Membrane Immunofluorescence of Transplanted Leukaemic Spleen Cells with H-2 Alloantisera*

| Leukaemic target cell | Serum | % unstained | FI | *P* |
|---|---|---|---|---|
| HIV | $H\text{-}2^d$ anti $H\text{-}2^b$ | 84 | 0·06 | NS |
| | NMS | 89 | | |
| MNUI | $H\text{-}2^d$ anti $H\text{-}2^b$ | 87 | 0·11 | <0·005 |
| | NMS | 96 | | |
| HII | $H\text{-}2^d$ anti $H\text{-}2^b$ | 89 | 0·06 | <0·025 |
| | NMS | 95 | | |
| RI | $H\text{-}2^d$ anti $H\text{-}2^b$ | 91 | 0·04 | NS |
| | NMS | 95 | | |
| AI | $H\text{-}2^d$ anti $H\text{-}2^b$ | 78 | 0·11 | <0·0005 |
| | NMS | 97 | | |
| CV | $H\text{-}2^d$ anti $H\text{-}2^b$ | 91 | 0·09 | <0·001 |
| | NMS | 100 | | |
| P388 | $H\text{-}2^b$ anti $H\text{-}2^d$ | 81 | 0·04 | NS |
| | NMS | 84 | | |

mice pretreated with viable allogeneic normal lymphoid cells were tested against the same 8 transplanted leukaemias and their reactivity compared with that of sera from non-immune mice of the appropriate strain.

In tests with leukaemias HIV, RI and P388 there was no difference in the number of stained cells between the H-2 alloantiserum and control serum (Table VI). FI values ranged from 0·04 to 0·06 (mean $0 \cdot 05 \pm 0 \cdot 01$). Significant degrees of staining with alloantisera compared with normal mouse serum were obtained against

FIG.—Relative absorption capacities of transplanted leukaemia (RI and CV) cells and normal spleen cells for Balb/c anti-C57Bl (H-2$^d$ anti H-2$^b$) alloantiserum. Upper figure (a) ○———○, ○— — —○, RI cells (duplicate absorptions) ●———● normal spleen cells. Lower figure (b) ○———○, ○— — —○, CV cells (duplicate absorptions) ●———● normal spleen cells.

the remaining leukaemias (MNUI, HII, AI and CV) although the FIs were low (range, 0·06–0·11, mean 0·09±0·02) (Table VI). By contrast, the same alloantisera stained normal lymphoid cells from mice of the respective strains in 3 independent tests with FIs in the range 0·73–0·86 (mean 0·79±0·04).

*Absorption of alloantiserum.*—The ability of leukaemias RI and CV in comparison with normal C57Bl spleen cells to absorb alloantibody was evaluated in a series of tests. No correction was made for the approximately five-fold increase in size of the leukaemic cells compared with normal spleen cells.

Comparison of the profiles in Fig. 1a,b indicates that for a standard volume of reference antiserum (H-2$^d$ anti H-2$^b$) significantly greater numbers of leukaemic cells were required in both instances to reduce the FI compared with normal spleen cells, notwithstanding the substantially increased surface area of the leukaemic cells to which the alloantiserum was exposed.

*Detection of cell-surface antigens on primary leukaemias by indirect membrane immunofluorescence (IF)*

Lymphoid tissues from 9 primary BNU induced leukaemias arising in C57Bl mice were tested for H-2 alloantigens with Balb/c anti-C57Bl (H-2$^d$ anti H-2$^b$) antiserum (Table VII). Leukaemic spleens from all mice showed markedly diminished reactivity with the alloantiserum (mean FI, 0·08±0·08) compared with lymphoid cells from the spleens of normal untreated mice (mean FI 0·68±0·06). In one example only (LS3) the FI value was slightly elevated but this was insignificant compared with the normal spleen controls. Comparably, the reactivity of leukaemic thymuses (tested in 6 mice) to the alloantiserum was greatly reduced, FIs falling in the range −0·04—0·11 (mean FI 0·0± 0·08), compared with normal thymocytes against which the FIs were 0·51–0·72 (mean FI 0·60±0·11). However, lymph nodes from 2 leukaemic mice showed increased numbers of stained cells (FIs 0·38 and 0·31) compared with leukaemic thymuses and spleen, but were still diminished compared with normal lymph node cells, for which the FI values were 0·68 in separate tests.

*Capacity of leukaemia cells to evoke antibody production in allogeneic recipients*

Alloantisera produced in Balb/c mice following 2 injections of 10$^7$ Al, RI or CV leukaemia cells were tested against the immunizing leukaemia cells and normal

TABLE VII.—*Reactivity by Indirect Membrane Immunofluorescence of Primary C57Bl Leukaemias with H-2 Alloantiserum* ($H$-$2^d$ *anti* $H$-$2^b$)

| Mouse No. | Target cells | % Cells unstained | FI* |
|---|---|---|---|
| 1 | leukaemic spleen | 87 | 0·02 |
| | normal spleen | 22 | 0·73 |
| | leukaemic thymus | 90 | −0·10 |
| | normal thymus | 32 | 0·67 |
| | leukaemic lymph node | 55 | 0·38 |
| | normal lymph node | 28 | 0·68 |
| 2 | leukaemic spleen | 78 | 0·08 |
| | normal spleen | 30 | 0·64 |
| | leukaemic thymus | 86 | 0·10 |
| | normal thymus | 46 | 0·51 |
| 3 | leukaemic spleen | 67 | 0·22 |
| | normal spleen | 30 | 0·64 |
| | leukaemic thymus | 85 | 0·11 |
| | normal thymus | 46 | 0·51 |
| | leukaemic lymph node | 69 | 0·31 |
| | normal lymph node | 28 | 0·68 |
| 4 | leukaemic spleen | 86 | 0·01 |
| | normal spleen | 30 | 0·64 |
| | leukaemic thymus | 98 | −0·04 |
| | normal thymus | 46 | 0·51 |
| 5 | leukaemic spleen | 71 | 0·18 |
| | normal spleen | 30 | 0·64 |
| | leukaemic thymus | 93 | −0·04 |
| | normal thymus | 46 | 0·51 |
| 6 | leukaemic spleen | 99 | 0 |
| | normal spleen | 30 | 0·64 |
| | leukaemic thymus | 98 | −0·01 |
| | normal thymus | 23 | 0·72 |
| 7 | leukaemic spleen | 86 | 0·05 |
| | normal spleen | 30 | 0·64 |
| 8 | leukaemic spleen | 99 | 0·02 |
| | normal spleen | 30 | 0·64 |
| 9 | leukaemic spleen | 89 | 0·11 |
| | normal spleen | 17 | 0·76 |

* Calculated with respect to NMS value

TABLE VIII.—*Reactivity by Indirect Membrane Immunofluorescence of Allogeneic Leukaemia Antisera with the Immunizing Leukaemia Cells and Normal Lymphoid Cells*

| | | Serum FI versus:- | | | |
|---|---|---|---|---|---|
| Target cells | Immunization | AI | RI | CV | Mean FI (±S.E.) |
| Leukaemic | 2 × 10⁷ cells i.p. | 0·05 | 0·02 | 0 | 0·02±0·03 |
| Normal | | 0·53 | 0·66 | 0·60 | 0·60±0·07 |
| Leukaemic | 4 × 10⁷ cells i.p. | 0·01 | 0·03 | 0·06 | 0·03±0·03 |
| Normal | | 0·86 | 0·70 | 0·78 | 0·78±0·08 |

spleen cells respectively. Without exception, when leukaemia cells were used as targets the proportion of unstained cells exposed to the leukaemia antisera did not differ from those treated with normal mouse serum, FIs ranging from 0 to 0·05 (mean $0{\cdot}02 \pm 0{\cdot}03$) (TableVIII). By contrast, each of these antisera reacted strongly with normal spleen cells to give FIs from 0·53 to 0·66 (mean $0{\cdot}60 \pm 0{\cdot}07$). The capacity of the allogeneic leukaemia antisera to stain cells of the immunizing leukaemias could not be increased by hyperimmunization (mean FI $0{\cdot}03 \pm 0{\cdot}03$). However, higher FI values ranging from 0·70 to 0·86 (mean FI $0.78 \pm 0{\cdot}08$) were obtained against normal spleen cells with these antisera.

### DISCUSSION

The disclosure of significant differences in expression of antigenic components on the surface of leukaemic and normal murine lymphoid cells is dependent on reliable and sensitive methods for antigen detection. In the study of the cell-surface antigens of leukaemic cells two techniques have principally been used, *viz.* complement dependent immune cytolysis and membrane immunofluorescence (IF). Neither of these methods is without disadvantages; immune cytolysis is a complex process dependent not only on antibody binding but also on complement activation as well as properties related to membrane vulnerability and ability of cells to repair membrane damage (Lerner, Oldstone and Cooper, 1971). Membrane immunofluorescence, on the other hand, is a sensitive and technically facile method although evaluation of results depends on visual observations that are not readily quantified. Whilst recognizing these limitations, the results presented in this paper were obtained using the latter technique and purport to show that, in comparison with normal lymphoid cells, there is gross modification of surface antigen expression on radiation and chemically-induced leukaemic cells. These conclusions are based principally on the findings that H-2 alloantisera capable of detecting H-2 antigens on the surface of normal cells from different lymphoid organs with an appreciable degree of sensitivity failed to reveal comparable antibody binding to the tumour cells; and also the demonstration of residual antibody reactivity against normal lymphoid cells following absorption of reference alloantisera with graded numbers of leukaemic cells. In some instances, such cell surface changes may be related to serial *in vivo* passage, or in the case of P388, to maintenance in tissue culture. While this possibility cannot be excluded for the transplanted leukaemias studied, a close association with leukaemogenesis is strongly implied by the finding that similar modification of H-2 antigen expression was a feature common to 9 BNU induced *primary* leukaemias.

There are several interpretations of the apparent diminution in antibody binding sites on the surface of primary and transplanted leukaemia cells which are not necessarily mutually exclusive. First, H-2 antigens are quantitatively deleted from the cell surface; second, the increased volume of leukaemic cells compared with normal lymphoid cells brings about spatial redistribution of antibody binding sites to a density below the threshold level of detection by IF; and third, H-2 antigen expression is modified by other factors such as masking.

Antigens dependent on the H-2 chromosome region in mice comprise many distinct specificities. The marked reduction in staining with multispecific H-2 antisera would not be inconsistent with an overall reduction of all the specificities or with disappearance of those to which antibodies were primarily directed. Since the IF test as employed in this study is capable of monitoring only gross changes in H-2 components, no distinction can be made between these possibilities. Precedents exist for both: qualitative disappearance of certain antigens on malignant cells has been claimed (Rubin *et al.*, 1970;

Seigler *et al.*, 1971) while in other cases a general diminution, without detectable qualitative loss, has been reported (Haywood and McKhann, 1971).

No information is available on the minimum antigen density required for detection of cell surface components by IF. It is possible that the five-fold increase in surface area of the leukaemic cells compared with normal lymphoid cells might reduce the capability of the test. However, this was not supported by absorption studies where exposure of antiserum to a surface area of leukaemic cells approximately 5 times that of normal spleen cells failed to abolish the reactivity of antibody with normal lymphoid cells.

The possibility that H-2 antigens on the surface of leukaemic cells are nonexposed or present in cryptic form merits consideration. Treatment of certain cell types with neuraminidase reveals otherwise concealed histocompatibility antigens, as demonstrated by increased susceptibility of cells to the appropriate antibody *in vitro* and abrogation of allogeneic transplantability *in vivo* (Sanford, 1967; Schlesinger and Amos, 1971; Schlesinger and Gottesfeld, 1971). Masking phenomena other than sialic acid coating are known to exist (Friberg and Lilliehöök, 1973). In several mouse tumour systems failure to detect H-2 antigens, shown by cell fractionation procedures to be localized exclusively in the cell membrane, was attributed to steric hindrance of cell surface determinants in the leukaemic cells (Molnar, Klein and Friberg, 1973). Masking of antigen, as distinct from deletion, possibly accounts more adequately for the humoral antibody response generated against H-2 antigens in allogeneic mice. However, the degree to which unavoidable contamination of the immunizing leukaemia cell preparations with a small minority of normal lymphoid cells contributes to this antibody induction is difficult to assess quantitatively and should not be disregarded.

Most experimentally induced leukaemias express neoantigens detectable serologically or by *in vivo* transplantation procedures (Pasternak, 1969). In the present study only 2 of 5 transplanted leukaemias provided any evidence of immunogenicity, and this was weak. The association of this property with P388 leukaemia cells in particular is obscure. This might reflect histocompatibility differences between tumour and host, culminating from a long history of transplantation and *in vitro* culture, or antigenic conversion (Stück, Old and Boyse, 1964), an interpretation consistent with the large numbers of virus particles associated with this neoplasm (T. D. Allen, personal communication).

Attempts to induce resistance against 3 other transplanted leukaemias in this study were unsuccessful. Several possibilities might account for this apparent lack of neoantigenicity, *e.g.* the deficiency may be intrinsic or a consequence of immuno selection. However, since some murine lymphomata exist for which the $LD_{50}$ in syngeneic recipients is only a few cells, the requirement of $\sim 10^3$ cells to ensure leukaemia development implies some resistance on the part of the host. This being so, failure to significantly augment this protection by immunization, other than in an apparently nonspecific way, or to induce synthesis of specific antibody, may be a reflection of the inadequacy of the immunization protocol rather than lack of immunogenicity. However, alternative methods of inducing resistance or raising antibody using mitomycin-C treated cells or subthreshold inocula have not proved significantly better (unpublished finding). These data would not therefore be inconconsistent with the hypothesis that failure to detect unequivocally tumour neoantigens on these leukaemias is related to complex cell surface changes affecting antigen expression as a whole.

### REFERENCES

AOKI, T., BOYSE, E. A., OLD, L. J., DE HARVEN, E., HAMMERLING, U. & WOOD, H. A. (1970) G (Gross) and H-2 Cell-Surface Antigens: Location on Gross Leukemia Cells by Electron Microscopy with Visually Labelled Antibody. *Proc. natn. Acad. Sci. U.S.A.*, **65**, 569.

AOKI, T. & TAKAHASHI, T. (1972) Viral and Cellular Surface Antigens of Murine Leukemias and Myelomas. Serological Analysis by Immunoelectron Microscopy. *J. exp. Med.*, **135**, 443.

BALDWIN, R. W. & GLAVES, D. (1972) Deletion of Liver-cell Surface Membrane Components from Aminoazo-dye-induced Rat Hepatomas. *Int. J. Cancer*, **9**, 76.

BOYSE, E. A. & OLD, L. J. (1969) Some aspects of Normal and Abnormal Cell Surface Genetics. *A. Rev. Genet.*, **3**, 269.

DAWE, C. J. & POTTER, M. (1957) Morphologic and Biologic Progression of a Lymphoid Neoplasm of the Mouse *in vivo* and *in vitro*. *Am. J. Path.*, **33**, 603.

DOLJANSKI, F. (1973) A New Look at the Cell Surface. In *Immunological Parameters of Host-tumour Relationships*, Vol. 2. D. W. Weiss. New York and London: Academic Press Inc. p. 47.

FOX, M. & GILBERT, C. W. (1966) Continuous Irradiation of a Murine Lymphoma Line P388F *in vitro*. *Int. J. radiat. Biol.*, **11**, 339.

FRIBERG, S. & LILLIEHÖÖK, B. (1973) Evidence for non-exposed H-2 Antigens in Immunoresistant Murine Tumour. *Nature, New Biol.*, **241**, 112.

HAYWOOD, G. R. & MCKHANN, C. F. (1971) Antigenic Specificities on Murine Sarcoma Cells. Reciprocal Relationship between Normal Transplantation Antigens (H-2) and Tumor-specific Immunogenicity. *J. exp. Med.*, **133**, 1171.

KLEIN, G. (1968) Tumor-specific Transplantation Antigens: G. H. A. Clowes Memorial Lecture. *Cancer Res.*, **28**, 625.

LERNER, R. A., OLDSTONE, M. B. & COOPER, N. R. (1971) Cell Cycle-dependent Immune Lysis of Moloney Virus-transformed Lymphocytes: Presence of Viral Antigen, Accessibility to Antibody and Complement Activation. *Proc. natn. Acad. Sci. U.S.A.*, **68**, 2584.

MÖLLER, G. (1961) Demonstration of Mouse Isoantigens at the Cellular Level by the Fluorescent Antibody Technique. *J. exp. Med.*, **114**, 415.

MOLNAR, J., KLEIN, G. & FRIBERG, S. JR (1973) Subcellular Localization of Murine Histocompatibility Antigens in Tumor Cells. *Transplantation*, **16**, 93.

MOTTA, R. & BRULEY, M. (1973) Quantitative Study of the Histocompatibility Antigens on the Surface of Normal and Leukemic Cells in Mice. I. Variations in the Expression of Groups of H-2 Specificities in Four Leukemias induced by 7, 12-dimethyl(α) anthracene. *Transplantation*, **15**, 22.

PASTERNAK, G. (1969) Antigens Induced by the Mouse Leukaemia Viruses. *Adv. Cancer Res.*, **12**, 1.

RUBIN, D. J., COLTEN, H. R., BORSOS, T. & RAPP, H. J. (1970) Antigenic Loss in a Transplantable, Chemically Induced Leukemia of C57BL/6 Mice. *J. natn. Cancer Inst.* **44**, 975.

SANFORD, B. H. (1967) An Alteration in Tumor Histocompatibility Induced by Neuraminidase. *Transplantation*, **5**, 1273.

SCHLESINGER, M. & AMOS, B. D. (1971) Effect of Neuraminidase on Serological Properties of Murine Lymphoid Cells. *Transplantn Proc.*, **3**, 895.

SCHLESINGER, M. & GOTTESFELD, S. (1971) The Effect of Neuraminidase on Expression of Cellular Antigens. *Transplantn Proc.*, **3**, 1151.

SEIGLER, H. F., KREMER, W. B., METZGAR, R. S., WARD, F. E., HAUNG, A. T. & AMOS, D. B. (1971) HL-A Antigenic Loss in Malignant Transformation. *J. natn. Cancer Inst.*, **46**, 577.

STOCKERT, E., OLD, L. J. & BOYSE, E. A. (1971) The $G_{IX}$ system. A Cell Surface Allo-antigen associated with Murine Leukaemia Virus; Implications regarding Chromosomal Integration of the Viral Genome. *J. exp. Med.*, **133**, 1334.

STÜCK, B., OLD, L. J. & BOYSE, E. A. (1964) Antigenic Conversion of Established Leukaemias by an Unrelated Leukaemogenic Virus. *Nature, Lond.*, **202**, 1016.